# MicroRNA-137 Drives Epigenetic Reprogramming in the Adult Amygdala and Behavioral Changes after Adolescent Alcohol Exposure

**DOI:** 10.1523/ENEURO.0401-19.2019

**Published:** 2019-11-22

**Authors:** Evan J. Kyzar, John Peyton Bohnsack, Huaibo Zhang, Subhash C. Pandey

**Affiliations:** 1 Center for Alcohol Research in Epigenetics, Department of Psychiatry, University of Illinois at Chicago, Chicago, IL 60622; 2 Jesse Brown Veterans Affairs Medical Center, Chicago, IL 60612; 3 Department of Anatomy and Cell Biology, University of Illinois at Chicago, Chicago, IL 60622

**Keywords:** adolescent alcohol, amygdala, anxiety, BDNF, epigenetics, microRNA-137

## Abstract

Adolescent binge drinking is a serious public health concern and a risk factor for alcohol use disorder (AUD) and comorbid anxiety in adulthood. Chromatin remodeling mediated by epigenetic enzymes including lysine-specific demethylase 1 (LSD1) due to adolescent alcohol exposure may play a role in adult psychopathology. The mechanism by which adolescent alcohol exposure mechanistically regulates epigenetic reprogramming and behavioral changes in adulthood is unknown. We investigated the role of microRNA-137 (miR-137), which is crucial for normal neurodevelopment and targets LSD1, in adolescent intermittent ethanol (AIE) exposure-induced anxiety-like and alcohol-drinking behaviors and related epigenetic reprogramming in the amygdala in adulthood. Adolescent rats were exposed to 2 g/kg ethanol (2 d on/off; AIE) or adolescent intermittent saline (AIS) during postnatal days (PND)28–PND41 and allowed to grow to adulthood for analysis of behavior, miRNA expression, and epigenetic measures in the amygdala. Interestingly, miR-137 was increased and its target genes *Lsd1* and *Lsd1 + 8a* were decreased in the AIE adult amygdala. Infusion of miR-137 antagomir directly into the central nucleus of the amygdala (CeA) rescues AIE-induced alcohol-drinking and anxiety-like behaviors via normalization of decreased *Lsd1* expression, decreased LSD1 occupancy, and decreased *Bdnf IV* expression due to increased H3K9 dimethylation in AIE adult rats. Further, concomitant *Lsd1* small interfering RNA (siRNA) infusion into the CeA prevents the miR-137-mediated reversal of AIE-induced adult anxiety and chromatin remodeling at the *Bdnf IV* promoter. These novel results highlight miR-137 as a potential therapeutic target for anxiety and AUD susceptibility after adolescent alcohol exposure in adulthood.

## Abstract (precis)

Adolescent binge drinking is a serious public health concern and a risk factor for alcohol use disorder (AUD) and comorbid anxiety in adulthood. Chromatin remodeling mediated by epigenetic enzymes including lysine-specific demethylase 1 (LSD1) due to adolescent alcohol exposure may play a role in adult psychopathology. The mechanism by which adolescent alcohol exposure mechanistically regulates epigenetic reprogramming and behavioral changes in adulthood is unknown.

## Significance Statement

Adolescent alcohol exposure is a serious public health problem and contributes to alcohol use and anxiety disorders later in life. In this study, we identify microRNA-137 (miR-137), a small non-coding RNA, in the central nucleus of the amygdala (CeA) as a crucial regulator of increased alcohol consumption and anxiety-like behavior in adult rats after adolescent intermittent ethanol (AIE) exposure. Inhibition of miR-137 in the CeA reverses increased alcohol intake and anxiety-like behavior, and this effect is mediated by lysine-specific demethylase 1 (LSD1), a miR-137 target gene that regulates epigenetic programming. Thus, we have identified miR-137 and its target LSD1 in the CeA that play a mechanistic role in the pathogenesis of increased adult anxiety and alcohol consumption after adolescent alcohol exposure.

## Introduction

Adolescence is an important developmental period for neuromaturation, as numerous biological changes including gene expression and synaptic remodeling in discrete brain regions inform typical adolescent behaviors like impulsivity and novelty-seeking (Keshavan et al., 2014; Spear, 2018). The long-term consequences of adolescent binge drinking also include increased risk of alcohol use disorder (AUD) and other psychiatric disorders including anxiety and depression (Swendsen et al., 1998; Chassin et al., 2002; Jennison, 2004). Physiologic changes in the developing brain are at least partly mediated by epigenetic mechanisms (Lister et al., 2013), which constitute changes in genomic architecture that alter gene expression without altering the underlying DNA sequence (Berger et al., 2009). Rodent models reveal that exposure to alcohol during the crucial developmental period of adolescence leads to increased anxiety-like behaviors, alcohol preference, and epigenetic changes in brain regions including the amygdala (Pascual et al., 2009; Pandey et al., 2015; Kyzar et al., 2016, 2017; Montesinos et al., 2016; Trantham-Davidson et al., 2017). The amygdala is a hub regulator of anxiety and negative affective states that plays a crucial role in maintaining and escalating addictive behaviors (Koob and Volkow, 2010; Pandey et al., 2017).

MicroRNAs (miRNAs) are small, non-coding RNAs that are ∼22–23 nucleotides long and function to negatively regulate mRNA stability and translation (Bartel, 2009). miRNAs play critical roles in neurodevelopment and dendritic spine outgrowth (Miska et al., 2004; Schratt et al., 2006; Im and Kenny, 2012), and contribute to the pathogenesis of psychiatric disorders (Lewohl et al., 2011; Muiños-Gimeno et al., 2011; Issler and Chen, 2015; Warnica et al., 2015). miRNAs interact with other epigenetic factors, such as histone modifications and DNA methylation, to tightly regulate gene expression in the brain in response to environmental stimuli (Kenny, 2014). Altered miRNA expression in specific brain circuits regulates alcohol-related behaviors (Tapocik et al., 2014; Darcq et al., 2015; Teppen et al., 2016), and miRNAs are potential biomarkers for the effects of developmental alcohol exposure (Balaraman et al., 2014).

While aspects of epigenetic regulation such as histone modifications and DNA methylation are altered in the adult amygdala following adolescent alcohol exposure (Pandey et al., 2015; Kyzar et al., 2017, 2019; Sakharkar et al., 2019), the contribution of amygdala miRNAs to chromatin remodeling and the observed phenotype of increased anxiety and alcohol consumption in adulthood after adolescent alcohol exposure remains unknown. We recently reported that lysine-specific demethylase 1 (LSD1; also known as KDM1A) is decreased in the adult amygdala, but not in the bed nucleus of the stria terminalis (BNST), after adolescent alcohol exposure in male rats, and this is associated with increased repressive H3K9 dimethylation (H3K9me2) levels particularly at crucial synaptic plasticity related genes such as brain-derived neurotrophic factor (*Bdnf*) and unchanged levels of H3K4me2 (Kyzar et al., 2017). Interestingly, microRNA-137 (miR-137) is the only miRNA empirically shown to target LSD1 and is implicated in psychiatric illness including schizophrenia (Sun et al., 2011; Schizophrenia Working Group of the Psychiatric Genomics Consortium, 2014; Mahmoudi and Cairns, 2017). However, miR-137 expression and its effect on epigenetic and behavioral outcomes in adulthood after adolescent alcohol exposure is not currently known. Therefore, we examined the effects of adolescent intermittent ethanol (AIE) on miR-137 and target gene expression. The mechanistic role of miR-137 in the regulation of LSD1-mediated epigenetic effects in the amygdala and anxiety-like and alcohol-drinking behaviors in adulthood after AIE was also investigated.

## Materials and Methods

### AIE exposure and behavioral studies

All animal experiments were approved by the Institutional Animal Care and Use Committee at the University of Illinois at Chicago and complied with National Institutes of Health guidelines for animal use. Timed-pregnant Sprague Dawley (SD) rats or dams with pups were ordered from a vendor (Harlan Laboratories). Rats were kept on a 12/12 h light/dark cycle and provided chow and water *ad libitum*. Rats were weaned at postnatal day (PND)21 and were exposed to either AIE or adolescent intermittent saline (AIS; 0.9% NaCl) beginning at PND28. As described previously (Pandey et al., 2015; Kokare et al., 2017; Kyzar et al., 2017, 2019), male rats were randomly assigned to groups and exposed to either ethanol (2 g/kg, i.p.; AIE) or volume-matched saline (AIS) on a 2-d on/off schedule from PND28 to PND41, for a total of eight injections. Rats were allowed to grow to adulthood (PND92–PND98) and amygdaloid tissues (predominantly containing central and medial nucleus of amygdala but also a small portion of basolateral amygdala) were used for biochemical measures (RNA expression, and chromatin occupancy). A subset of both AIS and AIE male rats was exposed to an acute challenge of 2 g/kg ethanol (intraperitoneal) or volume-matched saline in adulthood (PND101–PND102) prior to sacrifice 1 h after injection and extraction of the amygdala for the measurement of miR-137 levels as described below.

### Central nucleus of the amygdala (CeA) cannulation and infusion of miR-137 antagomir

Separate cohorts of AIS and AIE male rats were bilaterally cannulated directly targeting the CeA under anesthesia with inhaled isoflurane during adulthood (PND >80). Rats were allowed at least one week to fully recover from surgery. Animals were placed in a stereotaxic apparatus and bilaterally implanted with CMA/11 guide cannulae (CMA Microdialysis) as previously published (Moonat et al., 2013). The coordinates for the CeA were 2.5 mm posterior to bregma, 4.2 mm lateral to the bregma, and 5.1 mm ventral from the point of cannula entry at the skull surface. Rats were singly-housed after surgery and monitored daily for recovery. Following recovery, rats were bilaterally infused in the CeA using a Hamilton syringe (Hamilton) to inject 400 pmol (in 0.5 μl) of an antisense locked nucleic acid (LNA) antagomir construct specific to miR-137 (Exiqon), scrambled LNA (Negative Control B; Exiqon), or vehicle (iFect solution; Neuromics). The sequence of the miR-137 antagomir construct is G*T*A*T*T*C*T*T*A*A*G*C*A*A*T, with asterisks representing phosphorothioated nucleotides. Rats were infused twice per day (9 A.M. and 5 P.M.) for 2 d, similar to our previous studies using antagomir technology (Teppen et al., 2016). Another batch of AIS- and AIE-exposed animals were exposed to both miR-137 antagomir and *Lsd1* small interfering RNA (siRNA; or control siRNA). miR-137 antagomir was administered similarly with 400 pmol of antagomir given bilaterally twice per day for 2 d (9 A.M. and 5 P.M.), and *Lsd1* siRNA (0.5 μg/0.5 μl per side) was concomitantly given once along with the 9 A.M. infusion of miR-137 antagomir on the second day of infusions. The sequence of *Lsd1* siRNA is sense; 5'-CAACGUCCUCAAUAAUAAATT-3', antisense; 5'-UUUAUUAUUGAGGACGUUGAA-3' (QIAGEN), and the negative control siRNA used was obtained from QIAGEN (AllStars Negative Control siRNA). On the third day, animals were tested for behavior in the elevated plus maze (EPM) and their amygdala tissue was immediately collected for downstream biochemical processing. The timing of antagomir and siRNA infusion was chosen based on the effects of similar constructs on the behavior of adult rats in prior studies (Moonat et al., 2013; Teppen et al., 2016; Kyzar et al., 2019). A separate batch of rats was generated for infusion of miR-137 antagomir into the CeA to examine the effect on alcohol consumption measured using a two-bottle free-choice alcohol-drinking paradigm, as described below. Cannulae placement was confirmed for all rats at the time of dissection of amygdaloid tissues for biochemical measurements and was further confirmed in some rats using Nissl staining as described previously (Pandey et al., 2006; Zhang et al., 2010).

### Behavioral studies

For measurement of anxiety-like behavior, animals were tested in the EPM as described previously (Pandey et al., 2015; Kyzar et al., 2017, 2019). The batch of adult rats exposed only to miR-137 antagomir were tested 16 h after last miR-137 antagomir (or scrambled/vehicle) infusion at PND110–PND111. The batch of adult rats exposed to both miR-137 antagomir and *Lsd1* siRNA were tested 16 h after last miR-137 antagomir (or scrambled) infusion and 24 h after last *Lsd1* siRNA (or scrambled) infusion at PND118–PND119. The percentage of open arm entries and the percentage of time spent in the open arm represent anxiety-related endpoints, while the number of closed arm entries is a measure of general activity (File, 1993).

A cohort of AIS and AIE male adult rats were exposed to miR-137 antagomir or vehicle during a two-bottle free choice voluntary ethanol drinking paradigm, similar to our previous studies (Pandey et al., 2015). Following cannulation surgery on PND95–PND96, AIS and AIE adult rats were single-housed and after recovery received water in two bottles until no significant bottle preference was detected, which took two weeks. Subsequently, rats received water in one bottle and increasing concentrations (w/v) of ethanol (3% of ethanol for 3 d, 7% of ethanol for 3 d and 9% of ethanol for 9 d) in the other bottle. AIS and AIE rats were then bilaterally infused with miR-137 antagomir or vehicle directly into the CeA twice per day (9 A.M. and 5 P.M.) on the fourth and fifth day of 9% alcohol intake, and we continued to monitor their 9% ethanol and water intake. After miR-137 antagomir or vehicle treatment was terminated, rats continued to receive 9% ethanol in one bottle and water in the other bottle to determine whether the alcohol preference would return to pre-treatment levels. All rats received fresh bottles each day in the evening between 5 and 6 P.M., and ethanol and water intake was measured (ml/d) at this time. The position of the bottles was exchanged every day to avoid preference formation for bottle position.

### miRNA measurement by qPCR

TaqMan mature microRNA qPCR assays (Thermo Fisher Scientific) were purchased for miR-137 (for primer sequences, see Table 1), and qPCR was performed according to the assay protocol using TaqMan Universal PCR Master Mix (no UNG) as described previously (Teppen et al., 2016) using the Mx3000P qPCR system (Agilent Technologies) and MxPro software. *U6* was used as a reference gene for miRNA qPCR analysis. The Ct value of *U6* was subtracted from the Ct value of target miRNA, and fold changes were calculated using the 2-ΔΔCT method (Livak and Schmittgen, 2001; Kyzar et al., 2017). Data are expressed as fold change of AIS control rats.

**Table 1.** Primers sets used for miRNA analysis (using TaqMan probe assays), qPCR analysis of mRNA transcripts, and ChIP assay using qPCR

Primer name	Sequence
TaqMan miRNA primers	
miR-137-3p	UUAUUGCUUAAGAAUACGCGUAG
U6	AGAAGATTAGCATGGCCCCT
mRNA primers	
*Lsd1* forward	CGCCACGGTCTTATCAACTT
*Lsd1* reverse	GCCAGAAACACCTGAGCCTA
*Lsd1 + 8a* forward	GAGGAAATCCCATGGCTGT
*Lsd1 + 8a* reverse	GGAACCTTGACAGTGTCAGCTT
*Bdnf IV* mRNA forward	TCTCACTGAAGGCGTGCGAGTATT
*Bdnf IV* mRNA reverse	TGGTGGCCGATATGTACTCCTGTT
*Hprt1* forward	TCCTCAGACCGCTTTTCCCGC
*Hprt1* reverse	TCATCATCACTAATCACGACGCTGG
ChIP qPCR primers	
*Bdnf IV* promoter forward	GTTCGCTAGGACTGGAAGTGG
*Bdnf IV* promoter reverse	CCTCTGCCTCGAAATAGACAC

### mRNA quantification by qPCR

Quantification of mRNA was conducted using reverse transcription PCR and primers specifically designed for rat mRNAs *Lsd1*, *Lsd1 + 8a*, and *Bdnf* exon IV (Table 1). RNA was isolated from amygdala tissue as described previously (Kyzar et al., 2017, 2019) and then reverse transcribed in duplicate using mixed random primers and MuLV reverse transcriptase (Life Technologies). Quantitative real-time PCR was performed using either the Mx3000P qPCR system (Agilent Technologies) and SYBR Green master mix (Fermentas) or a CFX Connect qPCR system with iQ SYBR SuperMix (Bio-Rad). Data were analyzed using the 2-ΔΔCT method (Livak and Schmittgen, 2001). *Hprt1* was used as a reference gene (Kyzar et al., 2017, 2019). Data are represented as fold change compared to control AIS groups.

### Chromatin immunoprecipitation (ChIP) assay

The ChIP assay was used to measure occupancy of H3K4me2, H3K9me2, and LSD1 proteins (Kyzar et al., 2017, 2019; Bohnsack et al., 2019). Amygdala tissue was fixed in methanol-free formaldehyde, and chromatin was then sheared to 200- to 500-bp fragments by sonication with the Covaris ME220 (Covaris). The chromatin complex was immunoprecipitated with specific antibodies to H3K9me2 (Abcam ab1220, RRID: AB_449854, 3 μg/sample), LSD1 (Abcam ab17721, RRID: AB_443964, 3 μg/sample), and H3K4me2 (Abcam ab32356, RRID: AB_732924, 3 μg/sample). The precipitated DNA was quantified by qPCR using a CFX Connect qPCR system with iQ SYBR SuperMix (Bio-Rad) and primers designed to specific genomic regions (Table 1). Rabbit IgG (Millipore; catalog #NI01; 3 μg/sample) was used as a negative control antibody but did not amplify any genomic region tested in qPCR. Input DNA Ct value was subtracted from the Ct value of each respective sample. The 2-ΔΔCT method (Livak and Schmittgen, 2001) was used to determine the fold change compared to AIS control groups.

### Experimental design and statistical analysis

Two group experiments involving AIS- and AIE-exposed rats were analyzed using an independent sample two-tailed (Student’s) *t* test. Experiments involving animals exposed to AIS or AIE in adolescence followed by an acute ethanol or saline challenge in adulthood were analyzed with two-way ANOVA. This was followed by *post hoc* comparison using Tukey’s test. For miR-137 antagomir experiments involving five groups, one-way ANOVA was employed to test for differences between groups followed by a *post hoc* Tukey’s test. For miR-137 antagomir experiments in the two-bottle free choice alcohol-drinking paradigm, two-way repeated measures ANOVA was performed. All experiments were performed in biological replicates indicated by the “*n*” value indicated in each figure legend. At least two technical replicates were averaged for each biochemical measure. We did not perform power analysis to predetermine group size and instead based sample sizes on previous experiments performed using similar protocols and effect sizes in the lab. Data are presented as mean ± SEM. Significance for all experiments was set at *p* < 0.05. Exact *p* values are reported up to *p* < 0.001.

## Results

### AIE exposure leads to increased miR-137 and decreased *Lsd1* expression in the amygdala

**Figure 1.**
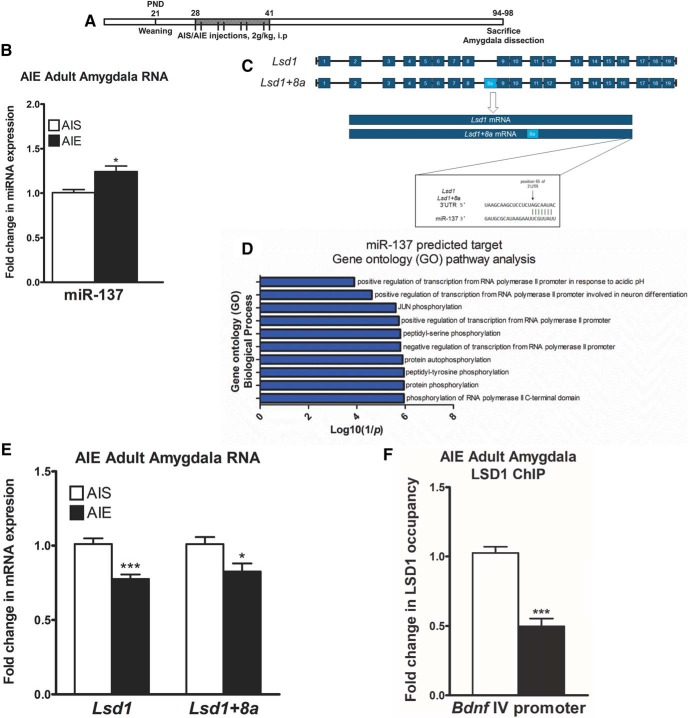
AIE exposure increases miR-137 expression in the male adult amygdala. ***A***, Schematic of AIE or adolescent intermittent saline (AIS) exposure followed by behavioral analysis and amygdala tissue collection in adulthood. ***B***, qPCR measurement of miR-137 in the amygdala of AIS and AIE adult rats (*n* = 5–6, *p < 0.05 by Student’s *t* test). ***C***, Schematic of miR-137 targeting of *Lsd1* and the neuron-specific splice variant *Lsd1 + 8a* mRNA. ***D***, GO analysis performed with Enrichr for enrichment of GO Biological Process terms using a list of bioinformatically-predicted miR-137 target genes (Extended Data Fig. 1-1). The top ten enriched GO terms are expressed as a log10(1/*p* value). ***E***, qPCR measurement of *Lsd1* and *Lsd1 + 8a* mRNA in the amygdala of AIS and AIE adult rats (*n* = 5–6, *p < 0.05, ***p < 0.001 by Student’s *t* test). ***F***, ChIP analysis of LSD1 occupancy of the *Bdnf* exon IV promoter region in the amygdala of AIS and AIE adult rats (*n* = 5, ***p < 0.001 by Student’s *t* test). Data expressed as mean ± SEM.

We examined effects of AIE exposure (Fig. 1*A*) on RNA levels of miR-137 in the adult amygdala. It was found that AIE exposure increased miR-137 expression (*t*_(10)_ = –3.07, *p* = 0.013 by unpaired *t* test) in the amygdala of AIE adult rats (PND94; Fig. 1*B*). miR-137 is a brain-enriched miRNA (Landgraf et al., 2007) that is crucial for neuronal development. Gene ontology (GO) analysis of predicted miR-137 target genes, performed using the Enrichr software (Kuleshov et al., 2016), reveals five of the top ten overrepresented pathways involve transcriptional regulation and contain the epigenetic enzyme LSD1 (Fig. 1*C*,*D*; Extended Data Fig. 1-1), and bioinformatics analysis reveals a well-conserved miR-137 binding site in the 3’UTR of *Lsd1* (Fig. 1*C*). We observed decreased expression of *Lsd1* (*t*_(9)_ = 4.91, *p* < 0.001 by unpaired *t* test) and *Lsd1 + 8a* (*t*_(9)_ = 2.49, *p* = 0.034 by unpaired *t* test) mRNA in the amygdala of AIE adult rats compared to AIS adult rats (Fig. 1*E*). We then investigated the binding of LSD1 to the promoter of *Bdnf* exon IV and found decreased LSD1 binding to the *Bdnf IV* promoter (*t*_(8)_ = 7.31, *p* < 0.001 unpaired *t* test) in the AIE adult amygdala compared to AIS rats (Fig. 1*F*). Notably, we have previously shown that BDNF protein and *Bdnf IV* mRNA levels are decreased in the amygdala of adult AIE rats compared to adult AIS rats (Pandey et al., 2015), suggesting that decreased LSD1 may be involved in the regulation of BDNF expression after AIE in adulthood.

10.1523/ENEURO.0401-19.2019.f1-1 Extended Data Figure 1-1 Bioinformatic analysis of miR-137 target genes. miRNA target predictions for miR-137 in the rat were obtained from TargetScan (www.targetscan.org). The resulting gene target list was then analyzed using Enrichr (https://amp.pharm.mssm.edu/Enrichr/) specifically for Biological Process GO terms. The top ten GO Biological Process terms are shown here and sorted by the combined score as calculated in Enrichr, which is obtained by multiplying the log-transformed *p* value and *z* score. KDM1A, also known as LSD1, was used for validation in our functional study of inhibition of miR-137 by antagomir infusion into CeA. Download Figure 1-1, DOCX file.

### miR-137 inhibition in the CeA rescues AIE-induced anxiety-like behavior

**Figure 2.**
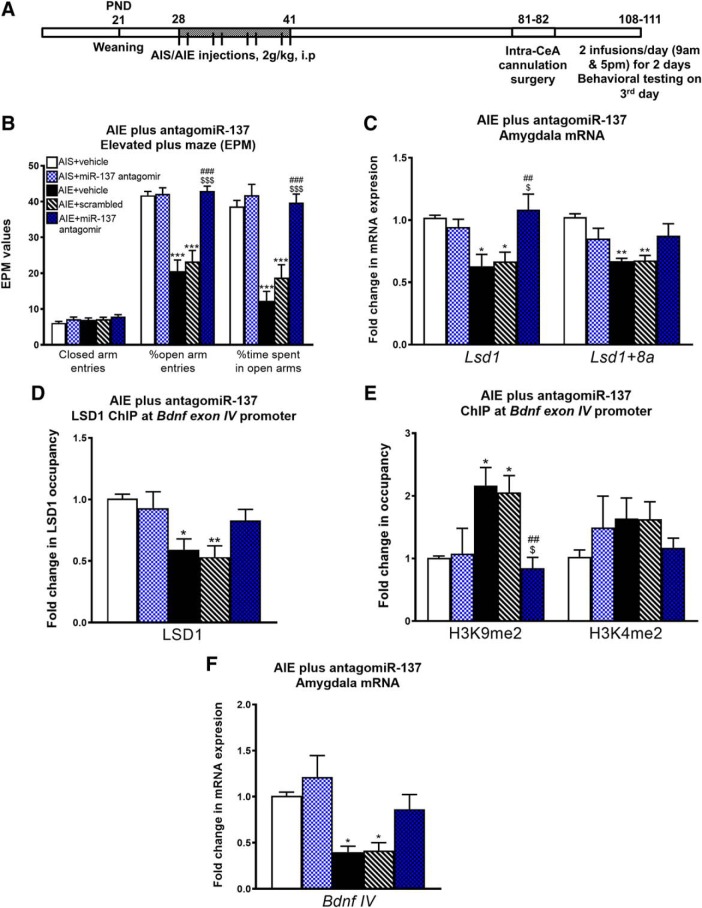
miR-137 antagomir infusion directly into the CeA reverses AIE-induced anxiety-like behaviors and target mRNA expression. ***A***, Schematic of AIE or adolescent intermittent saline (AIS) exposure followed by cannulation surgery and infusion of an antagomir specific to miR-137, scrambled antagomir, or vehicle directly into the CeA of adult male rats. ***B***, Bar diagram showing anxiety-like behaviors (measured in the EPM test) in AIS and AIE adult rats infused with miR-137 antagomir, scrambled antagomir, or vehicle (iFect solution) directly into the CeA (*n* = 6–7, ***p < 0.001 vs AIS+ vehicle, ###*p* < 0.001 vs AIE+ vehicle, $$$*p* < 0.001 vs AIE+ scrambled by one-way ANOVA followed by Tukey’s *post hoc* test). ***C***, qPCR analysis of the miR-137 target genes *Lsd1* and *Lsd1 + 8a* mRNA in the amygdala of AIS and AIE adult rats infused with miR-137 antagomir, scrambled antagomir, or vehicle directly into the CeA (*n* = 6–7, *p < 0.05 vs AIS+ vehicle, **p < 0.01 vs AIS+ vehicle, ##*p* < 0.01 vs AIE+ vehicle, $*p* < 0.05 vs AIE+ scrambled by one-way ANOVA followed by Tukey’s *post hoc* test). ***D***, ChIP analysis of LSD1 occupancy of the *Bdnf* exon IV promoter region in the amygdala of AIS and AIE adult rats infused with miR-137 antagomir, scrambled antagomir, or vehicle into the CeA (*n* = 6–7, *p < 0.05, **p < 0.01 vs AIS+ vehicle by one-way ANOVA followed by Tukey’s *post hoc* test). ***E***, ChIP analysis of H3K9me2 and H3K4me2 occupancy of the *Bdnf* exon IV promoter region in the amygdala of AIS and AIE adult rats infused with miR-137 antagomir, scrambled antagomir, or vehicle into the CeA (*n* = 6–7, *p < 0.05 vs AIS+ vehicle, ##*p* < 0.01 vs AIE+ vehicle, $*p* < 0.05 vs AIE+ scrambled by one-way ANOVA followed by Tukey’s *post hoc* test). ***F***, qPCR analysis of *Bdnf* exon IV (*Bdnf IV*) mRNA in the amygdala of AIS- and AIE-exposed adult rats infused with miR-137 antagomir, scrambled antagomir, or vehicle (iFect solution) into the CeA (*n* = 6–7, *p < 0.05 vs AIS+ vehicle by one-way ANOVA followed by Tukey’s *post hoc* test). Data expressed as mean ± SEM.

We next explored if increased expression of miR-137 in the CeA is responsible for AIE-induced anxiety-like behaviors by examining the effects of direct inhibition of miR-137 in the CeA on anxiety-like behaviors in AIE adult rats (Fig. 2*A*). Interestingly, infusion of miR-137-specific antagomir, but not scrambled antagomir, directly in the CeA significantly attenuated AIE-induced anxiety-like behaviors (Fig. 2*B*), as one-way ANOVA revealed a group level effect for percentage of open arm entries (*F*_(4,28)_ = 25.8, *p* < 0.001) and time spent in the open arms (*F*_(4,28)_ = 27.7, *p* < 0.001) but no change in the number of closed arm entries. *Post hoc* analysis showed that AIE animals exposed to either vehicle (AIE+ vehicle) or control antagomir (AIE+ scrambled) showed significantly decreased percentage of open arm entries and time spent in the open arms compared to AIS adult rats infused with vehicle (*p* < 0.001 by Tukey’s *post hoc* test). AIE rats infused with miR-137 antagomir (AIE+ miR-137 antagomir) show significantly increased percentage in entries and time spent in the open arms compared to AIE+ vehicle and AIE+ scrambled groups (*p* < 0.001 by Tukey’s *post hoc* test) but not compared to AIS+ vehicle groups. AIS rats infused with the miR-137 antagomir (AIS+ miR-137 antagomir) do not show any significant differences from AIS+ vehicle rats in anxiety measures. These results suggest that infusion of miR-137 antagomir into the CeA was able to attenuate anxiety-like behaviors in AIE adult rats.

### miR-137 inhibition in the CeA rescues AIE-induced epigenetic changes

We next measured mRNA expression of miR-137 target genes in the amygdala of adult rats after AIS or AIE exposure and infusion of miR-137 antagomir directly in the CeA. One-way ANOVA revealed a group level effect on *Lsd1* mRNA (*F*_(4,27)_ = 6.16, *p* = 0.001) and *Lsd1 + 8a* mRNA (*F*_(4,27)_ = 7.00, *p* < 0.001). *Lsd1* mRNA is decreased in the amygdala in AIE+ vehicle (*p* = 0.021 by Tukey’s *post hoc* test) and AIE+ scrambled (*p* = 0.040 by Tukey’s *post hoc* test) adult rats compared to AIS+ vehicle control rats (Fig. 2*C*). After miR-137 antagomir infusion in AIE rats, *Lsd1* mRNA expression is increased compared to AIE+ vehicle (*p* = 0.005 by Tukey’s *post hoc* test) and AIE+ scrambled (*p* = 0.011 by Tukey’s *post hoc* test) rats but is not different from AIS+ vehicle control rats. Similarly, *Lsd1 + 8a* mRNA is decreased in the amygdala in AIE+ vehicle (*p* = 0.001 by Tukey’s *post hoc* test) and AIE+ scrambled (*p* = 0.002 by Tukey’s *post hoc* test) adult rats compared to AIS+ vehicle control rats (Fig. 2*C*). *Lsd1 + 8a* mRNA levels are not significantly different from AIS+ vehicle levels in the amygdala of AIE rats infused with miR-137 antagomir.

LSD1 is an important epigenetic regulator that can remove methyl groups from both H3K4me1/2 and H3K9me1/2, depending on the genomic context and the binding of various nuclear cofactors (Shi et al., 2004; Metzger et al., 2005). Additionally, the neuron-specific splice variant LSD1 + 8a preferentially acts to demethylate H3K9me1/2 (Laurent et al., 2015). We determined whether the transcriptional downregulation of *Lsd1*/*Lsd1 + 8a* and subsequent rescue by inhibition of miR-137 (Fig. 2*C*) was accompanied by alterations in chromatin dynamics at the *Bdnf* exon IV (*Bdnf IV*) promoter, mRNA levels of which are decreased in the AIE adult amygdala (Pandey et al., 2015; Kyzar et al., 2017, 2019). Occupancy of LSD1 (*F*_(4,27)_ = 5.30, *p* = 0.003 by one-way ANOVA) and H3K9me2 (*F*_(4,27)_ = 6.17, *p* = 0.001 by one-way ANOVA), but not H3K4me2, were altered in AIE adult rats infused with miR-137 antagomir in the CeA in adulthood (Fig. 2*D*,*E*). *Post hoc* analysis revealed a significant deficit in LSD1 binding to the *Bdnf IV* promoter region in adult AIE rats infused with vehicle (*p* = 0.022 by Tukey’s *post hoc* test) or scrambled antagomir (*p* = 0.007 by Tukey’s *post hoc* test) compared to AIS+ vehicle control rats, but no significant difference between the AIS+ vehicle and AIE+ miR-137 antagomir adult rats (Fig. 2*D*). H3K9me2 occupancy was increased in the amygdala of both the AIE+ vehicle (*p* = 0.023 by Tukey’s *post hoc* test) and AIE+ scrambled (*p* = 0.046 by Tukey’s *post hoc* test) adult rats compared to AIS+ vehicle control rats (Fig. 2*E*). AIE+ miR-137 antagomir rats showed levels of H3K9me2 occupancy at *Bdnf IV* that were not different from AIS+ vehicle control rats and were significantly lower compared to both AIE+ vehicle (*p* = 0.007 by Tukey’s *post hoc* test) and AIE+ scrambled (*p* = 0.015 by Tukey’s *post hoc* test) adult rats.

We next determined whether the effect of altered LSD1 and H3K9me2 occupancy on *Bdnf IV* mRNA expression. *Bdnf IV* mRNA levels were significantly altered between groups (*F*_(4,28)_ = 7.47, *p* < 0.001 by one-way ANOVA) in AIS or AIE adult rats infused with either vehicle, scrambled antagomir, or miR-137 antagomir in the CeA. *Bdnf IV* mRNA is decreased in the amygdala in AIE+ vehicle (*p* = 0.016 by Tukey’s *post hoc* test) and AIE+ scrambled (*p* = 0.026 by Tukey’s *post hoc* test) adult rats compared to AIS+ vehicle control rats (Fig. 2*F*), but *Bdnf IV* mRNA levels are not significantly different between AIS+ vehicle and AIE+ miR-137 antagomir rats. These results suggest that infusion of miR-137 antagomir into the CeA was able to correct the deficits in *Bdnf IV* mRNA expression due to normalization of decreased LSD1 expression and the associated increase in H3K9me2 occupancy at the *Bdnf IV* promoter in AIE adult rats.

### Increased miR-137 expression in the CeA regulates anxiety-like behaviors and epigenetic changes after AIE in adulthood via LSD1

**Figure 3.**
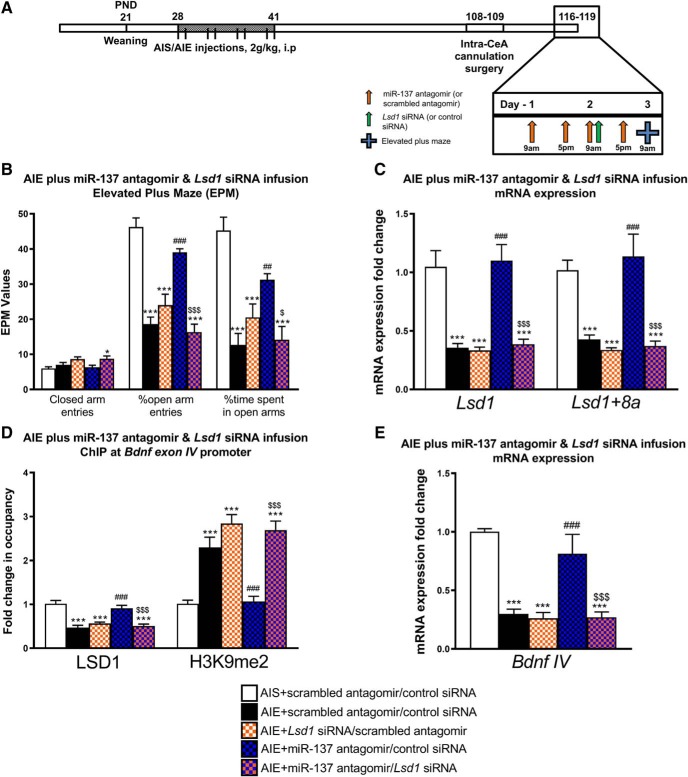
*Lsd1* siRNA infusion into the CeA prevents reversal of AIE-induced behavioral and epigenetic changes by miR-137 antagomir. ***A***, Schematic of AIE or adolescent intermittent saline (AIS) exposure followed by cannulation surgery and infusion of miR-137 antagomir or scrambled antagomir along with *Lsd1* siRNA or control siRNA into the CeA during adulthood. ***B***, Anxiety-like behavioral analysis in the EPM test in AIS and AIE adult rats infused with miR-137 antagomir or scrambled antagomir along with *Lsd1* siRNA or control siRNA directly into the CeA (*n* = 6–7, *p < 0.05, ***p < 0.001 vs AIS+ scrambled antagomir/control siRNA, ##*p* < 0.01, ###*p* < 0.001 vs AIE+ scrambled antagomir/control siRNA, $*p* < 0.05, $$$*p* < 0.001 vs AIE+ miR-137 antagomir/control siRNA by one-way ANOVA followed by Tukey’s *post hoc* test). Data expressed as mean ± SEM. ***C***, qPCR analysis of *Lsd1* and *Lsd1 + 8a* mRNA in the amygdala of AIS and AIE rats infused with miR-137 antagomir or scrambled antagomir along with *Lsd1* siRNA or scrambled siRNA directly into the CeA (*n* = 6–7, ***p < 0.001 vs AIS+ scrambled antagomir/control siRNA, ###*p* < 0.001 vs AIE+ scrambled antagomir/control siRNA, $$$*p* < 0.001 vs AIE+ miR-137 antagomir/control siRNA by one-way ANOVA followed by Tukey’s *post hoc* test). Data expressed as mean ± SEM. ***D***, ChIP analysis of LSD1 and H3K9me2 occupancy of the *Bdnf* exon IV promoter region in the amygdala of AIS and AIE adult rats infused with miR-137 antagomir or scrambled antagomir along with *Lsd1* siRNA or control siRNA directly into the CeA (*n* = 6–7, ***p < 0.001 vs AIS+ scrambled antagomir/control siRNA, ###*p* < 0.001 vs AIE+ scrambled antagomir/control siRNA, $$$*p* < 0.001 vs AIE+ miR-137 antagomir/control siRNA by one-way ANOVA followed by Tukey’s *post hoc* test). Data expressed as mean ± SEM. ***E***, qPCR analysis of *Bdnf IV* mRNA in the amygdala of AIS and AIE adult rats infused with miR-137 antagomir or scrambled antagomir along with *Lsd1* siRNA or control siRNA directly into the CeA (*n* = 6–7, ***p < 0.001 vs AIS+ scrambled antagomir/control siRNA, ###*p* < 0.001 vs AIE+ scrambled antagomir/control siRNA, $$$*p* < 0.001 vs AIE+ miR-137 antagomir/control siRNA by one-way ANOVA followed by Tukey’s *post hoc* test). Data expressed as mean ± SEM.

miR-137 has numerous verified target genes other than LSD1 (Mahmoudi and Cairns, 2017), and we therefore investigated the direct role of LSD1 in the miR-137 antagomir-mediated reversal of anxiety phenotype and epigenetic changes induced by AIE in adulthood. For this purpose, we cannulated AIS and AIE adult rats targeting the CeA and allowed them to recover. Subsequently, miR-137 antagomir infusion in the CeA was performed as shown in Figure 3*A* except *Lsd1* siRNA was co-infused once with the miR-137 antagomir on the third infusion (of four total antagomir infusions), 24 h before behavioral testing in the EPM and subsequent amygdala dissection for biochemical measures (Fig. 3*A*). This experiment allowed us to determine whether *Lsd1* mRNA rescue was crucial to the reversal of AIE-induced behavioral and epigenetic changes.

Concurrent inhibition of *Lsd1* by siRNA in the CeA was sufficient to prevent miR-137 antagomir-mediated rescue of the anxiety-like behaviors seen in the EPM in AIE adult rats (Fig. 3*B*), as one-way ANOVA revealed a group level effect for percentage of open arm entries (*F*_(4,27)_ = 32.8, *p* < 0.001), time spent in the open arms (*F*_(4,27)_ = 15.7, *p* < 0.001), and number of closed arm entries (*F*_(4,27)_ = 3.77, *p* = 0.015). *Post hoc* analysis showed that AIE rats infused with control siRNA and scrambled antagomir controls (AIE+ scrambled antagomir/control siRNA) show decreased time spent in the open arms and decreased percentage of entries into the open arms compared to AIS infused with scrambled antagomir and control siRNA rats (*p* < 0.001 by Tukey’s *post hoc* test). Decreased time spent in the open arms and decreased percentage of entries into the open arms, indicative of anxiety-like behavior, was also observed in AIE rats but was not modulated by *Lsd1* siRNA and scrambled antagomir infusion into CeA (AIE+ *Lsd1* siRNA/scrambled; *p* < 0.001 by Tukey’s *post hoc* test). AIE+ miR-137 antagomir/control siRNA infused adult rats showed increased time spent in the open arms (*p* = 0.005 by Tukey’s *post hoc* test) and percentage of entries into the open arms (*p* < 0.001 by Tukey’s *post hoc* test) compared to AIE+ scrambled antagomir/control siRNA rats, indicating a rescue of anxiety-like behavior as shown previously in Figure 2*B*. Interestingly, infusion with *Lsd1* siRNA together with miR-137 antagomir in AIE rats (AIE+ miR-137 antagomir/*Lsd1* siRNA) was able to block the miR-137 antagomir-mediated rescue of anxiety-like behaviors as indicated by decreased time spent in the open arms (*p* = 0.011 by Tukey’s *post hoc* test) and decreased percentage of entries into the open arms (*p* < 0.001 by Tukey’s *post hoc* test) compared to AIE+ miR-137 antagomir/control siRNA rats. AIE+ miR-137 antagomir/*Lsd1* siRNA rats also displayed increased closed arm entries compared to AIS control rats (*p* = 0.047 by Tukey’s *post hoc* test).

We found that co-infusion of *Lsd1* siRNA prevented the miR-137 antagomir-related normalization of epigenetic alterations in the AIE adult amygdala. One-way ANOVA revealed a group level effect on miR-137 target genes *Lsd1* mRNA (*F*_(4,27)_ = 20.1, *p* < 0.001) and *Lsd1 + 8a* mRNA (*F*_(4,27)_ = 17.2, *p* < 0.001). *Lsd1* and *Lsd1 + 8a* show decreased mRNA expression in AIE+ scrambled antagomir/control siRNA and AIE+ *Lsd1* siRNA/scrambled antagomir rats compared to AIS controls, and this decrease returns to baseline-like levels in AIE+ miR-137 antagomir/control siRNA rats (*p* < 0.001 by Tukey’s *post hoc* test). However, AIE+ miR-137 antagomir/*Lsd1* siRNA rats continue to display significantly decreased *Lsd1* and *Lsd1 + 8a* mRNA levels compared to both AIS+ antagomir scrambled/control siRNA and AIE+ miR-137 antagomir/control siRNA rats (*p* < 0.001 by Tukey’s *post hoc* test). We observed a similar pattern for LSD1 occupancy by ChIP at the *Bdnf IV* promoter (*F*_(4,27)_ = 20.8, *p* < 0.001 by one-way ANOVA; Fig. 3*D*), where AIE+ scrambled antagomir/control siRNA and AIE+ scrambled antagomir/*Lsd1* siRNA show decreased LSD1 occupancy that is rescued in the AIE+ miR-137 antagomir/control siRNA rats which is prevented in AIE+ miR-137 antagomir/*Lsd1* siRNA-infused rats (*p* < 0.001 by Tukey’s *post hoc* test). We additionally observed altered H3K9me2 occupancy at the *Bdnf IV* promoter (*F*_(4,27)_ = 22.1, *p* < 0.001 by one-way ANOVA), with increased H3K9me2 occupancy in the amygdala of AIE+ scrambled antagomir/control siRNA and AIE+ scrambled antagomir/*Lsd1* siRNA rats compared to AIS adult rats infused with scrambled antagomir/control siRNA (*p* < 0.001 by Tukey’s *post hoc* test; Fig. 3*D*). H3K9me2 occupancy at *Bdnf IV* in the amygdala returns to control-like levels after miR-137 antagomir infusion into the CeA, but the normalization is prevented by co-infusion of *Lsd1* siRNA (*p* < 0.001 by Tukey’s *post hoc* test). Lastly, one-way ANOVA revealed a group level effect on *Bdnf IV* mRNA (*F*_(4,27)_ = 19.4, *p* < 0.001; Fig. 3*E*). We observed that *Bdnf IV* mRNA expression is decreased in the AIE+ scrambled antagomir/control siRNA and AIE+ scrambled antagomir/*Lsd1* siRNA groups, returns to control-like levels in the AIE+ miR-137 antagomir/control siRNA rats, and decreased compared to both the AIE+ miR-137 antagomir/control siRNA and AIS control rats in animals co-infused with *Lsd1* siRNA and miR-137 antagomir (*p* < 0.001 by Tukey’s *post hoc* test). These results suggest that targeting of *Lsd1* mRNA by miR-137 and subsequent chromatin remodeling in the CeA is crucial in AIE-induced anxiety-like behaviors in adulthood.

### miR-137 inhibition in the CeA rescues AIE-induced increases in voluntary ethanol consumption

**Figure 4.**
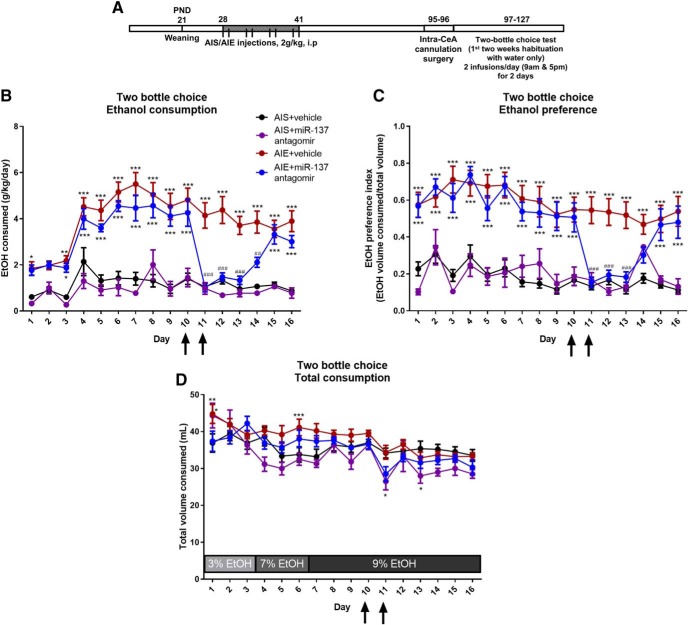
miR-137 antagomir infusion into the CeA reverses AIE-induced increases in adult alcohol consumption. ***A***, Schematic of AIE or adolescent intermittent saline (AIS) exposure followed by cannulation surgery and infusion of antagomir to miR-137 or vehicle (iFect solution) into the CeA during adulthood. ***B***, Measurement of ethanol consumption (g/kg/d) using two-bottle free choice paradigm. Arrows indicate days of miR-137 antagomir or vehicle infusion (twice per day for 2 d). Data expressed as mean ± SEM (*n* = 6–11, *p < 0.05, **p < 0.01, ***p < 0.001 vs AIS+ vehicle, ##*p* < 0.01, ###*p* < 0.001 vs AIE+ vehicle by two-way repeated measures ANOVA followed by Tukey’s *post hoc* test). ***C***, Measurement of ethanol preference using two-bottle free choice paradigm. Arrows indicate days of miR-137 antagomir or vehicle infusion (twice per day for 2 d). Data expressed as mean ± SEM (*n* = 6–11, ***p < 0.001 vs AIS+ vehicle, ###*p* < 0.001 vs AIE+ vehicle by two-way repeated measures ANOVA followed by Tukey’s *post hoc* test). ***D***, Total volume consumed (ml) in AIS and AIE rats exposed to either miR-137 antagomir or vehicle in a two-bottle free choice ethanol consumption task. Data are expressed as mean ± SEM (*n* = 6–11, *p < 0.05, **p < 0.01, ***p < 0.001 vs AIS+ vehicle by two-way repeated measures ANOVA followed by Tukey’s *post hoc* test). Arrows indicate days of miR-137 antagomir or vehicle infusion (twice per day for 2 d).

We next determined whether inhibition of miR-137 could also rescue increased alcohol consumption seen in AIE adult rats (Pandey et al., 2015). AIS and AIE adult rats were cannulated targeting the CeA and, after one week of recovery, habituated to drinking 9% ethanol (w/v). AIS and AIE rats were infused with either vehicle or miR-137 antagomir twice per day for 2 d (days 10–11) while their ethanol consumption was monitored (Fig. 4*A*). We observed significant effects of group (*F*_(3,495)_ = 52.1, *p* < 0.001 by two-way repeated measures ANOVA), day (*F*_(15,495)_ = 20.4, *p* < 0.001), and the interaction between group and day (*F*_(45,495)_ = 5.06, *p* < 0.001) on ethanol consumption (g/kg/d; Fig. 4*B*). We also found significant effects of group (*F*_(3,495)_ = 26.1, *p* < 0.001 by two-way repeated measures ANOVA), day (*F*_(15,495)_ = 13.4, *p* < 0.001), and the interaction between group and day (*F*_(45,495)_ = 4.31, *p* < 0.001) on ethanol preference index (Fig. 4*C*), and of group (*F*_(3,495)_ = 3.83, *p* = 0.019 by two-way repeated measures ANOVA), day (*F*_(15,495)_ = 14.6, *p* < 0.001), and the interaction between group and day (*F*_(45,495)_ = 2.17, *p* < 0.001) for total volume consumed (Fig. 4*D*). *Post hoc* analysis shows that AIE+ vehicle infused rats consume significantly more ethanol than AIS+ vehicle controls (g/kg/d) on days 1 (*p* = 0.040 by Tukey’s *post hoc* test) and 3–16 (*p* = 0.004-*p* < 0.001, Tukey’s *post hoc* test; Fig. 3*B*). AIE rats infused with miR-137 antagomir (AIE+ miR-137 antagomir) consume significantly more ethanol than AIS+ vehicle control rats on days 3–9 before miR-137 antagomir infusion, as well as on day 10 (*p* = 0.032- *p* < 0.001, Tukey’s *post hoc* test). However, AIE+ miR-137 antagomir infusion significantly decreased ethanol intake on the second day of miR-137 antagomir infusion (day 11; *p* < 0.001, Tukey’s *post hoc* test) and on days 12–14 after cessation of the infusions (*p* = 0.001- *p* < 0.001, Tukey’s *post hoc* test) as compared to AIE+ vehicle infused rats. AIE+ miR-137 antagomir infused rats resume drinking significantly more ethanol than AIS+ vehicle control rats 4 d following the cessation of last miR-137 antagomir infusion (*p* < 0.001 by Tukey’s *post hoc* test).

When evaluated for ethanol preference, the results are similar to total ethanol intake (Fig. 4*C*), with AIE+ vehicle rats showing increased ethanol preference compared to AIS+ vehicle controls (*p* < 0.001 by Tukey’s *post hoc* test) for the duration of the study. AIE+ miR-137 antagomir rats show increased ethanol preference compared to AIS+ vehicle rats on days 1–10 (*p* < 0.001 by Tukey’s *post hoc* test) before the miR-137 antagomir infusions are complete, and these rats then show decreased ethanol preference compared to AIE+ vehicle rats on days 11–13 (*p* < 0.001 by Tukey’s *post hoc* test). AIE+ miR-137 antagomir rats regain increased ethanol preference compared to AIS+ vehicle rats on days 15–16 (*p* < 0.001 by Tukey’s *post hoc* test), 4 d following the cessation of last miR-137 antagomir infusion. *Post hoc* differences in total volume consumed (Fig. 4*D*) during the 9% ethanol period of the study showed only decreased consumption in the AIS+ miR-137 antagomir group compared to the AIS+ vehicle rats on days 11 (*p* = 0.027 by Tukey’s *post hoc* test) and 13 (*p* = 0.040 by Tukey’s *post hoc* test). Importantly, the AIS+ miR-137 antagomir group showed no alterations in ethanol preference or ethanol consumption. These results suggest that AIE increases alcohol intake and is attenuated by the inhibition of miR-137 in the CeA of rats, suggesting that AIE-induced increases in miR-137 expression in the amygdala may drive increases in ethanol consumption in adulthood.

### Effects of acute ethanol challenge on the AIE-induced increase in RNA levels of miR-137 in the adult amygdala

**Figure 5.**
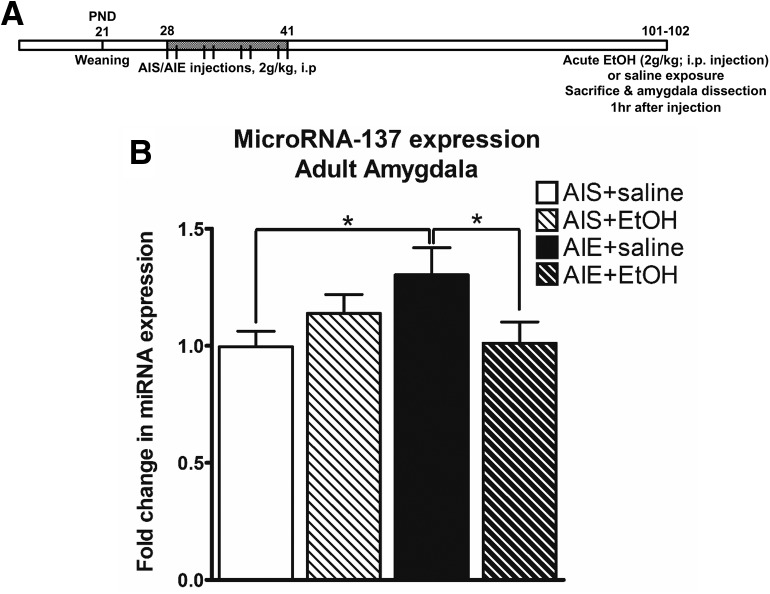
Effect of acute ethanol challenge in adulthood on AIE-induced increases in miR-137 levels in the amygdala of rats. ***A***, Schematic of AIE or adolescent intermittent saline (AIS) exposure followed by an acute challenge of ethanol (2 g/kg, i.p.) or volume-matched saline and sacrificed 1 h later. ***B***, qPCR measurement of miR-137 in the amygdala of AIS and AIE adult rats exposed to an acute challenge of ethanol or volume-matched saline. Data expressed as mean ± SEM (*n* = 6, *p < 0.05 by two-way ANOVA followed by Tukey’s *post hoc* test).

A previous study showed that the increased anxiety-like behavior seen in AIE adult rats, the decrease in *Lsd1 + 8a* mRNA in the AIE adult amygdala, and the increased H3K9me2 at the *Bdnf* exon IV promoter regions all return to control-like levels following an acute challenge with ethanol (2 g/kg) in adulthood (Kyzar et al., 2017; Fig. 5*A*). We extended these studies and examined if AIE rats may consume higher amounts of ethanol to inhibit miR-137 levels in the amygdala. We found that miR-137 expression in the amygdala (*F*_(1,20)_ = 5.82, *p* = 0.026 by two-way ANOVA: AIE treatment x adult acute ethanol interaction) is increased in AIE+ saline rats (*p* = 0.026 by Tukey’s *post hoc* test) and returns to control-like levels in the amygdala of AIE+ EtOH rats (*p* = 0.033 by Tukey’s *post hoc* test; Fig. 5*B*). These results suggest that ethanol exposure in AIE rats, but not in AIS rats, was able to inhibit the increased RNA levels of miR-137 in the amygdala and further supports the hypothesis that an increase in miR-137 levels in the amygdala is involved in AIE-induced alcohol-drinking behaviors in adulthood.

## Discussion

**Figure 6.**
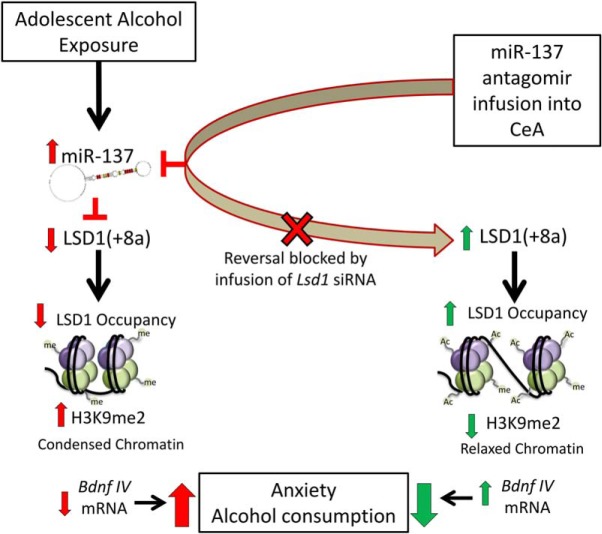
Conceptual model of the molecular and behavioral effects of AIE on miR-137 and LSD1-mediated chromatin remodeling in the adult amygdala. Adolescent intermittent exposure (AIE) leads to increased expression of miR-137, thereby decreasing expression of LSD1 and its neuron specific variant (LSD1 + 8a) in the adult rat amygdala. Increased expression of miR-137 is associated with decreased levels of LSD1 and increased levels of H3K9me2 at the *Bdnf* exon IV promoter, leading to decreased *Bdnf IV* mRNA expression and BDNF protein levels (Pandey et al., 2015). Blockade of the upregulation of miR-137 via antagomir in the CeA rescues these epigenetic modifications and deficits in *Bdn*f expression. miR-137 antagomir infusion in the CeA also attenuate the phenotypes of heightened anxiety and higher alcohol intake induced by AIE in adult rats. Concomitant inhibition of *Lsd1* by siRNA in the CeA blocks the miR-137 antagomir-mediated rescue of AIE-induced behavioral phenotypes and altered chromatin at the *Bdnf* exon IV promoter. These results identify miR-137 as an important epigenetic regulator in the CeA that mechanistically regulates AIE-induced anxiety-like and alcohol-drinking behaviors.

Our study shows that miR-137 is increased and its target *Lsd1* is decreased in the amygdala leading to epigenetic alterations contributing to the risk of anxiety-like and alcohol-drinking behaviors after AIE in adulthood. Inhibition of miR-137 in the CeA reversed AIE-associated anxiety and alcohol consumption phenotypes, as well as the altered LSD1-mediated chromatin remodeling at the *Bdnf IV* promoter. Concurrent inhibition of *Lsd1* mRNA in the CeA prevents the miR-137 antagomir-mediated rescue of AIE-associated behavioral and epigenetic changes. To our knowledge, this is the first investigation of miR-137 in the pathophysiology of alcohol-related behaviors, as well as the first demonstration of reversal of adolescent alcohol-related phenotypes in adulthood by targeting miR-137 in the CeA and mechanistically linking this to LSD1-mediated epigenetic reprogramming. Together, these data suggest that increased miR-137-mediated epigenetic reprogramming via decreased expression of LSD1 in the amygdala is involved in anxiety-like and alcohol-drinking behaviors after AIE in adulthood (Fig. 6).

The increased miR-137 seen in the AIE adult amygdala alters chromatin dynamics at the *Bdnf IV* promoter via downregulation of LSD1. We have previously shown that LSD1 protein levels are decreased in the CeA of AIE-exposed adult rats, and this decrease is associated with increased global levels of H3K9me2 protein but unchanged H3K4me2 protein levels (Kyzar et al., 2017). The current study shows that LSD1 protein occupancy is decreased and H3K9me2 is increased at the *Bdnf IV* promoter region, which is associated with decreased *Bdnf IV* mRNA expression in the amygdala of AIE adult rats. Interestingly, *BDNF* expression is decreased specifically in human postmortem amygdala of patients with AUD who began drinking before the age of 21, and levels of an antisense *BDNF* transcript (*BDNF-as*) are inversely correlated with *BDNF* expression (Bohnsack et al., 2019). Additionally, adult AIE rats show decreased lysine demethylase 6B (KDM6B) expression and altered KDM6B-mediated histone modifications at the activity-regulated cytoskeleton-associated (*Arc*) enhancer region in the amygdala, leading to decreased *Arc* enhancer RNA and mRNA expression (Kyzar et al., 2019). AIE appears to alter specific histone-modifying enzymes, such as LSD1 and KDM6B, that act on critical regulators of dendritic outgrowth and synaptic plasticity including BDNF and Arc. These important epigenetic regulators alter gene expression via multiple modalities including non-coding RNAs such as miR-137 and by the alteration of histone methylation and acetylation in the amygdala after AIE in adulthood.

We have reported decreased dendritic spine density and synaptophysin immunolabeling in the CeA and MeA following AIE exposure (Pandey et al., 2015; Kyzar et al., 2019), and miR-137 is a negative regulator of dendritic outgrowth and spine development by targeting the E3 ubiquitin-protein ligase *Mib1* (Smrt et al., 2010). miR-137 overexpression leads to deficits in synaptic transmission including decreased spontaneous neurotransmitter release accompanied by decreased synaptogenesis in mouse hippocampal neurons (He et al., 2018). Germline deletion of miR-137 results in embryonic lethality, but conditional haploinsufficiency of miR-137 in the nervous system leads to altered synaptic plasticity and behavior (Cheng et al., 2018). miR-137 is known to gradually increase across development (Hollins et al., 2014), and our results here suggest that adolescent alcohol exposure leads to an aberrant upregulation of miR-137 in adulthood that regulates epigenetic processes and adult psychopathology.

Previous studies have shown that the lasting behavioral effects seen after AIE are both strain-dependent and dependent on the method of alcohol exposure (Pascual et al., 2009; Gilpin et al., 2012; Alaux-Cantin et al., 2013; Ehlers et al., 2013; Varlinskaya et al., 2014; Pandey et al., 2015; Kyzar et al., 2017). Future studies should also probe different rat strains and exposure paradigms to determine the risk of specific genetic variants of miR-137 and its target genes. Interestingly, the deficits in BDNF and Arc in the adult amygdala in the AIE model (Pandey et al., 2015; Kyzar et al., 2019) used here are similar to molecular findings in human postmortem amygdala of AUD patients who started drinking before the age of 21 when compared with control subjects (Bohnsack et al., 2019). Downstream biochemical measures focused on the target gene LSD1 due to its known downregulation in the AIE adult amygdala (Kyzar et al., 2017) and also its enrichment in critical biological processes as determined by GO. It is important to point out that we cannot rule out the possible contributions of other verified miR-137 targets (Mahmoudi and Cairns, 2017) in the pathogenesis of AIE-induced anxiety and alcohol consumption, and future studies should investigate the role of these genes and validate additional targets. However, epigenetic effectors such as LSD1, as well as other proteins identified in our previous studies including KDM6B and HDAC2 (Pandey et al., 2015; Kyzar et al., 2019), are candidates for therapeutic intervention following early-life alcohol exposure due to the existence of pharmacologic agents targeting these proteins. Epigenetic proteins additionally bind to the genome and lead to ubiquitous downstream effects on transcription at many different loci, and as such they are likely involved in the altered expression of genes seen after adolescent alcohol exposure (Pandey et al., 2015, 2017; Kyzar et al., 2016, 2019). Finally, other miRNAs are likely involved in AIE-induced adult pathology, and these should be investigated in detail in future studies.

Another interesting finding of present study is that inhibition of miR-137 in the CeA of AIE adult rats was able to attenuate alcohol-drinking behaviors in a two-bottle choice drinking experiment. One hypothesis as to why miR-137 inhibition may be effective in decreasing drinking behaviors is that AIE rats drink higher amounts of ethanol to suppress miR-137 levels in the amygdala, thereby normalizing the AIE-induced deficits in LSD1 and subsequent epigenetic changes. We tested this possibility and found that acute ethanol challenge suppressed the AIE-induced increase in miR-137 levels in the amygdala, while the same dose of ethanol did not alter miR-137 levels in AIS rats. Earlier, we reported that acute ethanol challenge was also able to normalize *Lsd1 + 8a* mRNA levels and decrease repressive H3K9me2 occupancy of *Bdnf* exon IV in the amygdala of AIE, but not AIS, adult rats (Kyzar et al., 2017). Furthermore, acute ethanol challenge in AIE adult rats also attenuates anxiety-like behaviors (Kyzar et al., 2017, 2019). These data suggest that increased consumption of alcohol by AIE adult rats may act to normalize miR-137 levels, *Lsd1 + 8a* mRNA levels, H3K9me2 levels at *Bdnf* exon IV, and anxiety-like behaviors.

In summary, these results suggest that adolescent alcohol exposure causes enduring effects on miR-137 leading to downstream effects on chromatin remodeling through LSD1, heightened anxiety-like behavior, and higher alcohol consumption (Fig. 6). miR-137 antagomir infused directly into the CeA normalizes the aberrant AIE-induced behavioral and epigenetic effects, and this normalization is prevented by co-infusion of *Lsd1* siRNA. Our results highlight miR-137 and its target gene LSD1 in the amygdala as a potential therapeutic target for susceptibility to anxiety and AUDs in adulthood after adolescent ethanol exposure.

Acknowledgements: These studies were part of the PhD thesis work of EJK in the graduate college of University of Illinois at Chicago for his MD/PhD degree.

## Synthesis

Reviewing Editor: Kirill Martemyanov, The Scripps Research Institute Decisions are customarily a result of the Reviewing Editor and the peer reviewers coming together and discussing their recommendations until a consensus is reached. When revisions are invited, a fact-based synthesis statement explaining their decision and outlining what is needed to prepare a revision will be listed below. Note: If this manuscript was transferred from JNeurosci and a decision was made to accept the manuscript without peer review, a brief statement to this effect will instead be what is listed below.

The authors carefully addressed concerns of the previous review. The reviewer has no further comments or questions.
