# Supplementary material for: MicroRNA-137 Drives Epigenetic Reprogramming in the Adult Amygdala and Behavioral Changes after Adolescent Alcohol Exposure
Source: eNeuro. 2019 Nov 22;6(6):ENEURO.0401-19.2019. doi: 10.1523/ENEURO.0401-19.2019 (PMC6917896; doi:10.1523/ENEURO.0401-19.2019)
Supplement: Extended Data Figure 1-1 — Bioinformatic analysis of miR-137 target genes. miRNA target predictions for miR-137 in the rat were obtained from TargetScan (www.targetscan.org). The resulting gene target list was then analyzed using Enrichr (https://amp.pharm.mssm.edu/Enrichr/) specifically for Biological Process GO terms. The top ten GO Biological Process terms are shown here and sorted by the combined score as calculated in Enrichr, which is obtained by multiplying the log-transformed p value and z score. KDM1A, also known as LSD1, was used for validation in our functional study of inhibition of miR-137 by antagomir infusion into CeA. Download Figure 1-1, DOCX file. [file sup_enu-eN-NWR-0401-19-s02.docx]

**Extended Data Figure 1-1**. The data presented in table below supports Figure 1.

Bioinformatic analysis of miR-137 target genes.

| **Term** | **P-value** | **Adjusted P-value** | **Z-score** | **Combined Score** | **Genes** |
| --- | --- | --- | --- | --- | --- |
| positive regulation of transcription from RNA polymerase II promoter | 2.08E-09 | 1.82E-06 | -7.74352 | 154.7926 | **KDM1A**, MAML1, MYT1L, RORA, ARID4B, IKZF1, AHR, RBPJ, IKZF4, GRIP1, MEF2A, ACVR1, ZBTB38, SOX11, MITF, PIAS2, RUNX2, RFX7, TBL1XR1, PHIP, ERG, ZFPM2, BLZF1, NOTCH1, KMT2A, TWIST1, GLIS2, NEUROD1, NEUROD2, PRDM16, PPARGC1A, RREB1, PPARGC1B, ESRRA, EGR2, JAG1, BCL11B, SMARCA5, NFATC2, ESRRG, FOXN3, GDF6, BMP7, FLI1, TADA2B, MRGBP, SP1, AGO1, SP4, AGO2, JMY, TCF4, SP9, BRWD3, ZNF496, ARF4, KDM5B, CALCOCO1, PHF20, CHD1, ING5, MED14, ING2, TEAD1, NCK1, NCOA1, SS18, NCOA2, KLF12, NCOA3, TCF12, PROX1, KLF15, TOX3, KAT2B, SFPQ, MTF1, KIT, TET3, ZNF516, PLCB1, VGLL2, CREB5, NFIX, HLF, PLAG1, RNF4, CAMKK2, ERBB4, ATOH8, DAB2IP, WNT7A, ZNF76, KLF4, USF2, TBX3, KLF2, SKI, KLF7, TEF, NFIA, NFIB, ASXL2, NAA15, PAXBP1, SSBP2, SSBP3 |
| negative regulation of transcription from RNA polymerase II promoter | 1.36E-09 | 1.58E-06 | -7.21284 | 147.2598 | KDM5B, **KDM1A**, MYT1L, WWC1, OTUD7B, ARID4B, IKZF1, PRDM1, AHR, RBPJ, CHD1, BACH1, IKZF4, IKZF5, MECP2, MED14, NIPBL, TRPS1, MEF2A, PIAS4, KLF11, KLF12, ZBTB38, TCF12, NRG1, SOX11, MITF, PROX1, HIC1, RUNX2, FOXP1, SFPQ, RFX7, TBL1XR1, MTF1, KIT, ERG, ZMYND11, PLCB1, ZFPM2, BLZF1, NFIX, NOTCH1, CTBP1, TWIST1, PRICKLE1, ZBTB4, GLIS2, MTDH, SCRT2, NEUROD2, HMBOX1, ATXN1, BTAF1, NACC2, ATOH8, PRDM16, BEND3, ZNF148, FNIP1, ZBTB7A, E2F6, MTA3, RREB1, FNIP2, ZBTB18, KDM4A, DR1, SMURF2, MBD2, DAB2IP, SMARCA5, ZNF76, WWP2, FOXN3, KLF4, BMP7, FLI1, TBX3, SKI, TADA2B, KLF7, MRGBP, TEF, NFIA, SP4, COPS2, JMY, ZNF217, BCOR, MXD1, SP9, TRIM33, BRWD3, EZH2 |
| phosphorylation of RNA polymerase II C-terminal domain | 3.14E-10 | 1.13E-06 | -5.84228 | 127.8334 | KDM5B, GSK3B, **KDM1A,** MAML1, MYT1L, TESK2, ARID4B, AHR, CHD1, MED14, STK11, ACVR1, MAP4K2, KLF12, CSNK2A1, TCF12, LMTK2, OXSR1, PRKAB1, RUNX2, BCR, LATS2, RFX7, MTF1, MAPKAPK2, KIT, BMP2K, FAM20C, TSSK6, ALPK3, SIK1, TNIK, ERG, SIK2, BLZF1, PRKAA1, BRSK2, CTBP1, CAMKK2, NEUROD2, MAPK8, STK38, STK38L, FNIP2, PAK2, MARK1, SRPK2, MAP3K1, CSNK1A1, PLK2, SMARCA5, ZNF76, CDC42BPA, FLI1, TBX3, TADA2B, MAPK10, ICK, KLF7, SNRK, CDK6, MRGBP, TEF, SP4, JMY, PKN2, SP9, BRWD3 |
| positive regulation of transcription from RNA polymerase II promoter involved in neuron differentiation | 8.95E-08 | 2.41E-05 | -6.66112 | 108.1011 | ARF4, WNT2B, **KDM1A**, MAML1, PHF20, MYT1L, RORA, IKZF1, AHR, RBPJ, IKZF4, MED14, NCK1, MEF2A, ACVR1, NCOA1, SS18, NCOA2, ZBTB38, NCOA3, TCF12, SOX11, MITF, PROX1, KLF15, RUNX2, KAT2B, SFPQ, TBL1XR1, MTF1, IL1RAPL1, TET3, PHIP, ERG, ZFPM2, VGLL2, BRSK2, NFIX, NOTCH1, HLF, KMT2A, PLAG1, TWIST1, RNF4, GLIS2, NEUROD1, PPARGC1A, RREB1, PPARGC1B, ESRRA, EGR2, JAG1, BCL11B, DAB2IP, WNT7A, DCLK2, FZD8, ATP2B2, KLF4, USF2, KLF2, SKI, PTPRD, KLF7, NFIA, SP1, NFIB, AGO1, AGO2, ASXL2, TCF4, PAXBP1, SSBP2, SSBP3 |
| protein phosphorylation | 4.84E-10 | 1.13E-06 | -4.87693 | 104.6033 | CHRM3, GSK3B, MAML1, TESK2, NAGPA, STK11, SERP1, PPP4R2, AKT2, ACVR1, CSNK1G3, MAP4K2, EPHA7, CSNK2A1, LMTK2, OXSR1, PRKAB1, BCR, LATS2, PIK3CA, MAPKAPK2, BMP2K, FAM20C, TSSK6, ALPK3, SIK1, TNIK, ERG, TOP1, SIK2, PRKAA1, BRSK2, CTBP1, CAMKK2, MAPK8, STK38, STK38L, FNIP2, PAK2, MARK1, SRPK2, SLC35A1, MAP3K1, CSNK1A1, PLK2, WWP2, CDC42BPA, MAPK10, ICK, SNRK, CDK6, PKN2 |
| protein autophosphorylation | 7.38E-10 | 1.29E-06 | -4.80401 | 101.015 | GSK3B, PRKAA1, BRSK2, MAML1, CTBP1, SRC, TESK2, CAMKK2, STK11, MAPK8, ERBB4, STK38, STK38L, PAK2, FNIP2, MARK1, ACVR1, SRPK2, EPHA4, MAP4K2, PDGFRA, MAP3K1, CSNK2A1, EPHA8, CSNK1A1, PLK2, LMTK2, OXSR1, CDC42BPA, PRKAB1, MAPK10, BCR, ICK, SNRK, LATS2, CDK6, KIT, MAPKAPK2, BMP2K, ULK2, FAM20C, TSSK6, ALPK3, SIK1, PKN2, SIK2, TNIK, ERG |
| peptidyl-tyrosine phosphorylation | 3.42E-10 | 1.13E-06 | -4.5271 | 98.672 | GSK3B, PRKAA1, BRSK2, MAML1, CTBP1, SRC, TESK2, CAMKK2, STK11, MAPK8, ERBB4, STK38, STK38L, PAK2, FNIP2, MARK1, ACVR1, SRPK2, EPHA4, MAP4K2, PDGFRA, MAP3K1, CSNK2A1, CSNK1A1, PLK2, LMTK2, OXSR1, CDC42BPA, PRKAB1, MAPK10, BCR, ICK, SNRK, LATS2, CDK6, KIT, MAPKAPK2, BMP2K, FAM20C, TSSK6, ALPK3, SIK1, PKN2, SIK2, TNIK, ERG |
| peptidyl-serine phosphorylation | 1.27E-09 | 1.58E-06 | -4.5745 | 93.68908 | GSK3B, PRKAA1, BRSK2, MAML1, CTBP1, TESK2, CAMKK2, STK11, MAPK8, AKT2, STK38, STK38L, RICTOR, PAK2, FNIP2, MARK1, ACVR1, SRPK2, MAP4K2, CSNK1G3, MAP3K1, CSNK2A1, CSNK1A1, PLK2, DCLK2, LMTK2, OXSR1, CDC42BPA, PRKAB1, MAPK10, BCR, ICK, SNRK, LATS2, CDK6, SBK1, MAPKAPK2, BMP2K, FAM20C, TSSK6, ALPK3, SIK1, PKN2, SIK2, TNIK, ERG, TOP1 |
| JUN phosphorylation | 3.72E-09 | 2.45E-06 | -4.42182 | 85.82599 | GSK3B, PRKAA1, BRSK2, MAML1, CTBP1, TESK2, CRKL, CAMKK2, STK11, MAPK8, DUSP10, STK38, STK38L, PAK2, FNIP2, MARK1, ACVR1, SRPK2, MAP4K2, MAP2K4, MAP3K1, CSNK2A1, CSNK1A1, PLK2, LMTK2, OXSR1, CDC42BPA, PRKAB1, MAPK10, BCR, ICK, SNRK, LATS2, CDK6, MAPKAPK2, BMP2K, FAM20C, TSSK6, ALPK3, SIK1, PKN2, SIK2, TNIK, ERG |
| positive regulation of transcription from RNA polymerase II promoter in response to acidic pH | 1.64E-06 | 0.000127 | -6.319 | 84.17358 | ARF4, **KDM1A**, MAML1, PHF20, RORA, AHR, IKZF1, RBPJ, IKZF4, MED14, CHORDC1, NCK1, MEF2A, ACVR1, NCOA1, SS18, NCOA2, ZBTB38, NCOA3, TCF12, SOX11, MITF, PROX1, KLF15, KAT2B, SFPQ, TBL1XR1, MTF1, TET3, PHIP, ERG, ZFPM2, VGLL2, NFIX, NOTCH1, HLF, KMT2A, PLAG1, TWIST1, RNF4, GLIS2, NEUROD1, PPARGC1A, RREB1, PPARGC1B, ESRRA, EGR2, JAG1, BCL11B, DAB2IP, WNT7A, KLF4, USF2, KLF2, RAB11B, SKI, KLF7, NFIA, NFIB, SP1, AGO1, AGO2, ASXL2, TCF4, PAXBP1, SSBP2, SSBP3 |
